# Isolation and culture of smooth muscle cells from human acute type A aortic dissection

**DOI:** 10.1186/1749-8090-8-83

**Published:** 2013-04-12

**Authors:** Shuyang Lu, Xiaoning Sun, Tao Hong, Kai Song, Shouguo Yang, Chunsheng Wang

**Affiliations:** 1 Shanghai Institute of Cardiovascular Disease, Zhongshan Hospital, Fudan University, Fenglin Road 180, Xujiahui District, Shanghai, 200032, China; 2 Department of Cardiovascular Surgery, Zhongshan Hospital, Fudan University, Fenglin Road 180, Xujiahui District, Shanghai, 200032, China

**Keywords:** Type A aortic dissection (TAAD), Smooth muscle cells (SMCs), α-smooth muscle cell actin, Calponin

## Abstract

**Background:**

Acute type A aortic dissection (TAAD) is a life-threatening vascular disease. Smooth muscle cells (SMCs) are the main composition of aortic media and dysfunction of SMCs may lead to acute TAAD. The aim of this work was to investigate whether the SMCs of acute TAAD could be isolated and cultured for further research.

**Methods:**

TAAD tissues were obtained from acute TAAD patients who underwent emergent surgical treatment. A simple and economical technique of collagenase digestion method was used to isolate and culture human SMCs. Confocal laser scanning microscopy was applied to identify SMC phenotypes. Purity of isolated and cultured SMCs was analyzed with flow cytometry and fluorescence microscopy respectively.

**Results:**

The purity of isolated SMCs was 78.2%, including α-smooth muscle cell actin positive 13.9%, calponin positive 35.0% and double positive 29.3%. For cultured SMCs, abundant expression of α-smooth muscle cell actin was observed universally under fluorescence microscope. Confocal laser scanning microscope testified that cultured cells were double positive of α-smooth muscle actin and calponin.

**Conclusions:**

This is the first report of successful culture of SMCs isolated from human acute TAAD tissues. Living human SMCs of acute TAAD provides us with a new method for studying formation of acute TAAD.

## Background

Acute type A aortic dissection (TAAD) is the most disastrous cardiovascular disease. The International Registry of Acute Aortic Dissection (IRAD) shows that 70% patients would die within 1 week without intervention, 40% with medical treatment and 20% with surgical intervention only [1]. However, the mechanisms of acute TAAD formation are still unclear. Smooth muscle cells (SMCs) are the main component of aortic media and may participate in the formation of acute TAAD. Thus, isolation and culture of living human SMCs of TAAD will provide us with a new vector for research on mechanisms of acute TAAD in vitro.

Recently, SMCs have been cultured from several human tissues, placenta, bladder and intracranial aneurysms, umbilical cord [2-5]. However, the availability of SMCs from acute TAAD tissues for experimental studies has never been reported. In the present study, we reported the documented successful culture of SMCs from human acute TAAD tissues for the first time. SMCs phenotype was verified by surveying expression of α-smooth muscle actin and calponin. Purity of isolated and cultured SMCs was also analyzed.

## Methods

The study protocol was approved by the Committee for the Protection of Human Subjects at the Zhongshan Hospital, Fudan University. Informed consent was obtained from each patient involved in this study.

### Patient demographics and characteristics

Seven patients who underwent open aortic arch reconstruction for type A aortic dissection at Zhongshan Hospital (Shanghai, China) were included in the study. The mean age was 48.0 ± 14.1 years old (range 31–64), and 4 were male. More demographic and clinical data are shown in Table 1.

**Table 1:** Patient demographics and characteristics

**Patient no.**	**Age,y**	**Sex**	**Hypertension**	**DM**	**Respiratory dysfunction**	**Renal dysfunction**^**a**^	**SMCs culture**
1	31	M	+	-	-	-	success
2	51	F	-	-	-	-	success
3	38	M	-	-	-	-	success
4	61	M	+	+	-	-	failure
5	59	F	+	+	-	-	failure
6	32	M	+	-	-	-	success
7	64	F	+	-	+	+	failure

^a^ Serum creatinine > 2.0 mg/mL.

M, male; F, female; DM, diabetes mellitus.

### Isolation of SMCs from human acute TAAD tissues

**Figure 1:**
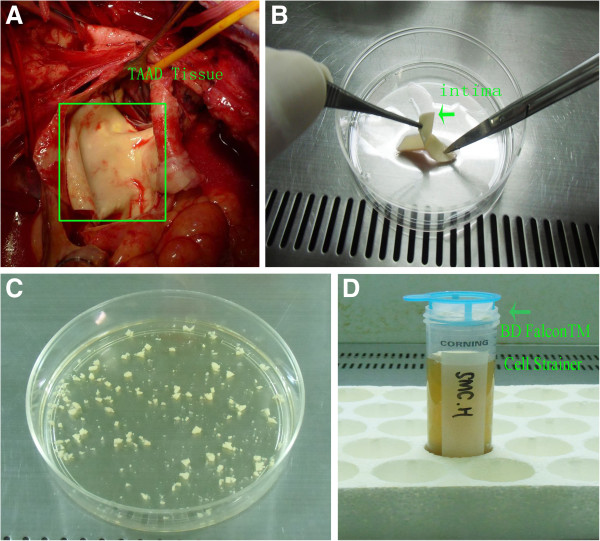
**Isolation of human SMCs from acute TAAD tissues. A:** acquisition of acute TAAD tissues; **B:** removing the intima carefully; **C:** cutting tunica media into 2-3 mm pieces and digesting with 0.1% type I collagenase; **D:** using BD Falcon™ Cell Strainer to remove the undigested explants and collect the cells.

TAAD vascular tissue was collected from patients undergoing emergent surgical treatment (Figure 1A) at Zhongshan Hospital. Then it was put into Dulbecco’s modified Eagle’s medium (DMEM) with penicillin/streptomycin (5 ml/500 ml) and transferred in super-clean bench. Under sterile conditions, vascular tissue was rinsed 3 times with phosphate-buffered saline (PBS) and intima was removed (Figure 1B). Tunica media were finely cut into 2-3 mm pieces in another 100 mm culture dish (Figure 1C). Four to 5 ml of 0.1% type I collagenase (Gibco, Invitrogen Corp) was added to the culture dish (Figure 1C). Then it was placed in an incubator for 1.5 to 2 hours at 37°C. Digestion media were collected and filtrated with BD Falcon™ Cell Strainer to remove the undigested explants, then centrifuged (1000 rpm, 5 minutes, 4°C). Above procedures were repeated for 3 times to acquire more cells. Acquired cells were used for purity analysis and cultured for further research.

### Purity analysis of isolated SMCs

For purity analysis of isolated SMCs, cells were pretreated with 4% paraformaldehyde for 6 to 8 hours and then permeated with 0.01% Triton 100/phosphate-buffered saline (PBS) for 10 minutes at room temperature. Cells were then sequentially incubated with primary antibodies (rabbit anti-α-smooth muscle cell actin, Epitomics Corp and mouse anti-calponin, Santa Cruz Biotechnology) at 4°C overnight and appropriate secondary antibodies (conjugated with PE, Abcam; fluorescein isothiocyanate, FITC, Jackson Immunoresearch Laboratories) on ice for 1 hour. After washing with PBS (5 minutes for 3 times) containing 0.5% bovine serum albumin (BSA), cells were resuspended in PBS and analyzed using FAC-ScanTM flow cytometer (Becton Dickinson, USA).

### Culture of isolated SMCs

Acquired cells were cultured in 25 ml culture bottles (5%CO_2_, 37°C). Media were removed and replaced with fresh media every 2 to 3 days. Cell morphology was observed daily with inverted light microscope. Cells started adhering within 36 hours and growing for 4 to 5 days. Cells grew to confluence within 2 weeks. Cells were placed in DMEM without serum for 24 hours to eliminate any contaminating cells, such as fobroblasts and endothelial cells, because these cells do not survive without serum [2]. Then, cells were cultured in DMEM with 10% fetal bovine serum according to standard protocols.

### Verification of SMCs Phenotype*-Confocal laser scanning microscopy*

Expression of α-smooth muscle cell actin and calponin were detected in SMCs by immunofluorescence staining method. Cells were cultured in glass bottom dishes (35 mm dish with 14 mm well), used for Confocal laser scanning microscope (CLSM) only. When the cells were growing at 50% confluence, they were washed and fixed with 4% paraformaldehyde for 15 minutes at room temperature, rinsed with PBS (5 minutes, 3 times) and permeated with 0.01%Triton 100 for 30 minutes. The cells were washed with PBS (5 minutes, 3 times) and blocking of nonspecific binding was performed by 5% bovine serum albumin (BSA) for 30 minutes. Then, the cells were incubated with rabbit anti-α-smooth muscle cell actin (1:200, Epitomics Corp) and mouse anti-calponin (1:200, Santa Cruz Biotechnology) overnight at 4°C. Corresponding secondary antibodies (conjugated with PE, Abcam; fluorescein isothiocyanate, FITC, Jackson Immunoresearch Laboratories) were applied in dilution 1:50 for 2 hours at room temperature.

### Immunofluorescence analysis

Expression of α-smooth muscle cell actin was analyzed in SMCs by immunofluorescence staining method. Cells were cultured in 6-well plate. Staining was executed as the methods stated above. Additionally, the nuclei of the cells were counterstained with 4, 6-diamidino-2-phenylindole (DAPI) for 5 minutes. Then stained cells were observed under common immunofluorescence microscope.

## Results

### Flow cytometry analysis

**Figure 2:**
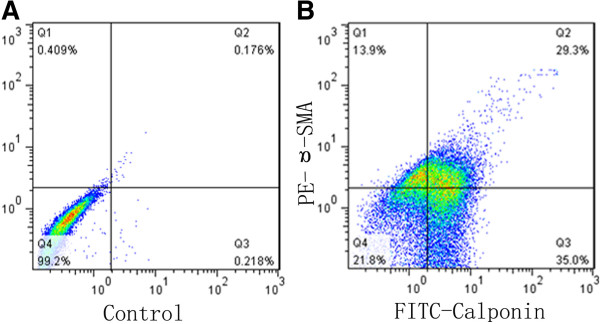
**Purity analysis of isolated SMCs with flow cytometry. A:** negative control; **B:** purity of isolated cells.

Purity of isolated cells was analyzed by FACScan™ flow cytometer. As shown in Figure 2B, the purity of primary isolated SMCs was 78.2%, including α-smooth muscle cell actin positive cells (13.9%), calponin positive cells (35.0%) and double positive cells (DP cells, 29.3%), while negative cells accounted for 21.8%. The negative control without treatment used for setting the scale was shown in Figure 2A.

### Cell cultures

**Figure 3:**
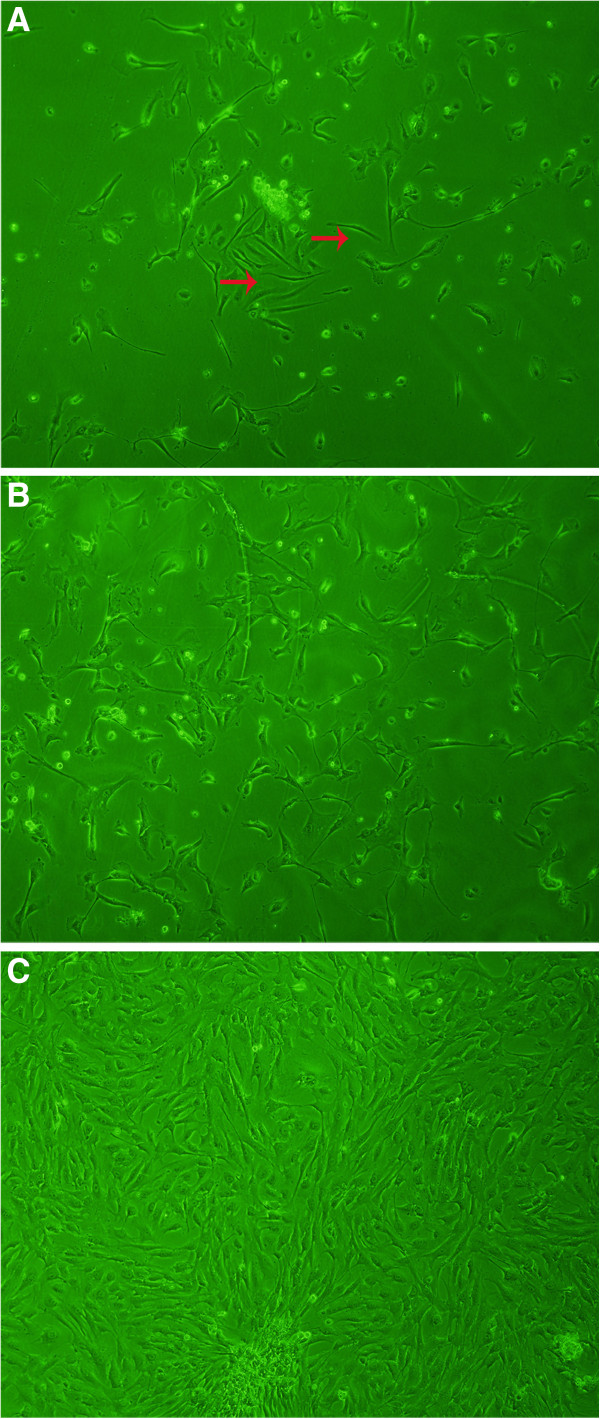
**Culture of isolated cells from acute TAAD tissues. A:** cells starting adhering within 36 hours and some cells were in parallel rows (red arrows); **B:** cells growing to about 30% within 4 to 5 days; **C:** cells growing into cells with the characteristic of “hills and valleys” within 2 weeks. Magnification is 200 × .

SMCs obtained by collagenase digestion method from acute TAAD tissues were observed starting adhering within 36 hours and initially slender in shape of some cells (red arrows) (Figure 3A) and growing to about 30% within 4 to 5 days (Figure 3B). Primary cultures of SMCs grew into cells with the characteristic of “hills and valleys” (typical of SMCs) within 2 weeks (Figure 3C).

### Expression of SMCs molecular markers

**Figure 4:**
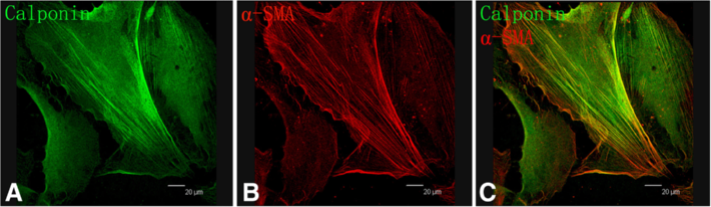
**Verification of SMCs Phenotypes. A:** cells showed uniform immunostaining of calponin; **B:** cells showed uniform filamentous of α-smooth muscle cell actin; **C:** cultured cells were double positive of calponin and α-smooth muscle cell actin. Magnification is 200×. Scale bar that can be seen in the right corner of these figures was equal to 20 um.

Uniform immunostaining for calponin treated with FITC (green) and α-smooth muscle actin treated with PE (red) were shown in Figure 4A and 4B respectively. Double marker-positive cells were shown in merged image (Figure 4C).

### Immunofluorescence analysis for cultured cells

**Figure 5:**
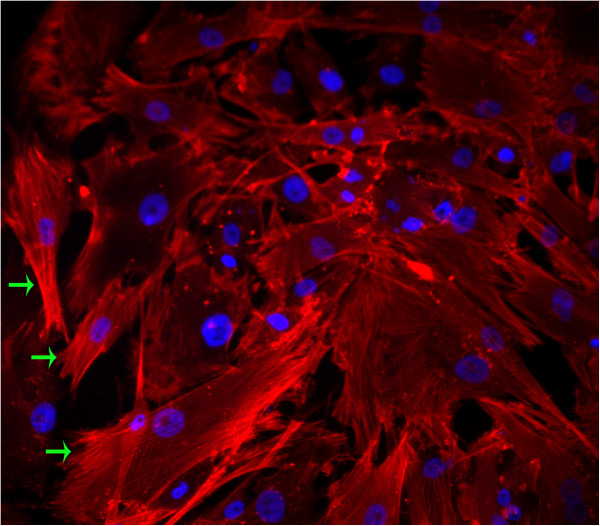
**Immunofluorescence analysis for cultured cells.** Uniform filamentous of α-smooth muscle actin (green arrows) can be observed universally. Magnification is 200 ×.

Abundant expression of α-smooth muscle cell actin treated with PE (red) was observed universally under fluorescence microscope. The nuclei of the cells were counterstained with DAPI (blue). The morphology of α-actin filaments could be clearly observed (green arrows) (Figure 5).

## Discussion

In the present study, we introduced a simple enzymatic digestion method to obtain human SMCs from acute TAAD tissues for in vitro studies. In explant culture, several weeks are needed to establish primary cultures and subculturing is required to acquire a large number of cells [2,4]. Additionally, isolation and culture of SMCs from acute TAAD tissues are very difficult for several reasons: firstly, acute TAAD patients are usually in acute inflammatory state according to our previous clinical observation and systematic inflammatory factors are adverse for the growth of isolated SMCs; secondly, tunica media (elastic fibers and collagen fibers) of human aorta are very compactible, which is hard for SMCs migrating from the explants; thirdly, the age of donor patients may be older [3], and the SMCs are in quiescence for a long time. As shown in Table 1, three cases in which SMCs were failed to be isolated and cultured were all with older age and hypertesion, two with diabetes mellitus and one with both respiratory and renal dysfunction. Since sample size was small, we need to testify these results in large samples. Due to above reasons, we applied collagenase digestion method in our research.

Several marker proteins were used to identify the origin and phenotype of our cultured cells. Actins are a category of highly conserved proteins universally expressed by all eukaryotic cells. They comprise, along with microtubules, a major component of the cytoskeleton and have been found to be expressed in at least six isomeric forms [6]. Amongst them, α-smooth muscle actin is a relatively specific gene of which the expression is relatively restricted to vascular smooth muscle cells [7]. Calponin is calcium- and actin-binding protein that regulates both the contractile machinery and cytoskeleton in SMCs [3,8]. In this paper, immunofluorescence staining observed with confocal laser scanning microscope indicated that the established primary acute TAAD tissue cell cultures were vascular SMCs.

In this study, purity of isolated cells was analyzed with flow cytometry, while purity of different cell lines was determined morphologically by phase-contrast microscopy and/or by immunofluorescence in previous studies [9]. Purity of primary isolated SMCs was 78.2%, indicating that intimal and adventitial connective tissues were completely removed. Reasons of DP cells and negative cells accounting for low percentage may be as follows: firstly, primary antibodies need to pass through the cell membrane permeabilized with Triton 100 before conjugating with target proteins; secondly, primary antibodies are indirectly conjugated with fluorescin coupled secondary antibodies, and the conjugation rate is low; thirdly, cells are in suspended state and in Brownian movement, which also increase the difficulty for conjugation.

For cultured cells, we use common fluorescence microscope to analyse the purity. α-smooth muscle actin positive cells are universally observed (see Figure 5). This may due to the further purificaiton of cultured cell. When growing in confluence, cells were placed in DMEM without serum for 24 hours which could, to some extent, eliminate some contaminating cells [2].

### Limitations

There are some inevitable limitations of the present study. First, the sample size is relatively small and only 7 cases in total were included. SMCs were just successful isolated and cultured in 4 cases. Secondly, it is quite difficult for us to get the healthy ascending vascular grafts to isolate the SMCs, therefore, there were no normal SMCs from healthy controls and no comparisons were carried out.

## Conclusions

We report this documented successful culture of SMCs isolated from human acute TAAD tissues for the first time. We expect these cells will provide us a new vector and valuable for in vitro research on mechanisms of acute TAAD.

## Competing interests

The authors declare that they have no competing interests.

## Authors’ contributions

SYL and XNS carried out the cell culture and immunostaining studies, and drafted the manuscript. TH participated in the flow cytometry analysis. KS carried out the samples collection. SGY and CSW conceived of the study, and participated in its design and coordination and helped to draft the manuscript. All authors read and approved the final manuscript.
